# Weak inclusion of the medical humanities in medical education: a qualitative study among Danish medical students

**DOI:** 10.1186/s12909-022-03723-x

**Published:** 2022-09-05

**Authors:** Elisabeth Assing Hvidt, Anne Ulsø, Cecilie Valentin Thorngreen, Jens Søndergaard, Christina Maar Andersen

**Affiliations:** 1 Research Unit of General Practice, Department of Public Health, University of Southern Denmark, J.B. Winsløwsvej 9 A, 5000 Odense, Denmark; 2 Department of Psychology, University of Southern Denmark, Odense, Denmark; 3 Steno Diabetes Center Odense, Odense University Hospital, Odense, Denmark

**Keywords:** Medical humanities, Empathy, Medical education, Denmark, Qualitative study

## Abstract

**Background:**

The modern medical education is predominantly grounded in the biomedical sciences. In recent years, medical humanities have been included into the medical curricula in many countries around the world one of the objectives being to promote patient-centred, empathic care by future physicians. Studies have been made of the impact of inclusion of medical humanities components within the medical curriculum. Although some results suggest increased empathy, others remain inconclusive. To gain insight into the depth, context, and impact of inclusion of the medical humanities for future physicians, this study aimed to explore Danish medical students’ understanding of and reflections on how the medical humanities relate to the medical education, including the clinic.

**Methods:**

We conducted a qualitative research study, involving semi-structured interviews with twenty-three Danish medical students across years of curriculum and medical schools. Interviews were recorded, transcribed verbatim and analyzed using Braun and Clarke’s thematic analysis.

**Results:**

The findings demonstrate the subordinate role of the medical humanities in the medical educational system. Students prioritize biomedical knowledge building in the preclinical curriculum, partly as a reaction to an unbalanced institutional inclusion of the medical humanities. Observing how structural empathy incentives are lacking in the clinical curriculum, the values inherent in the medical humanities are undermined.

**Conclusion:**

Danish medical students become part of an educational environment with lacking institutional conditions and structures to promote the strong inclusion of the medical humanities. A focus is therefore needed on the values, norms and structures of the medical educational systems that undermine a strong inclusion of the medical humanities into medical education.

## Introduction

The traditional modern medical curriculum is predominantly grounded in biomedical and clinical sciences, designed to equip future physicians with the skills and competencies to diagnose and cure diseases and to respond to scientific advances [1–4]. In the 1970s and ‘80 s, the psychiatrist George L. Engel discussed the need for medicine to adopt a more inclusive scientific model, the bio-psychosocial model, to avoid “producing” graduates deficient in personcentred- and empathic care [5, 6]. Contemporary bioethicists and medical humanists continue to support this call, pointing to the two-fold nature of medicine, biomedical and humanistic, and advocating for applying the same scientific rigor to the psychosocial model as customarily applied to the biomedical [4, 7–9]. Being attentive to dehumanizing tendencies in some areas of contemporary health care, and a burgeoning awareness of some of the shortcomings of the positivist influence on medical practice, medical educators have come to acknowledge the importance of exposing future medical practitioners to teaching subjects that inculcate “humaneness”, promoting empathy and compassion, and helping them develop reflective, critical, curious and creative faculties [10, 11].

The value of a humane health care is well documented: for example, studies show that empathy and compassion shown towards the patient in a clinical encounter improves the diagnostic process and -precision, patient satisfaction and -adherence, and thus increases treatment effectiveness and quality [12]. Not only for patients but also for physicians, and future physicians, empathy is of value: it protects clinicians from burnout and stress [13], from making medical errors and from malpractice complaints [14]. Moreover, studies show that high scores on empathy among medical students are associated with increased satisfaction with their education, lower levels of stress and burnout, higher ratings of overall clinical competences given by medical educational faculty, better interpretational skills assessed by patients and greater teamwork skills [15, 16].

Although the short- and longtime value of the medical humanities remain a debated and unresolved issue, including which “humanism” outcomes they may promote [17], results from reviews of the literature on empathy-enhancing educational interventions in undergraduate medical education suggest increasing empathy as effect [18–21] which is one out of many reasons why medical humanities teaching in medical curricula around the world have increased in recent years [8, 12, 22]. The meta-literature on the field (dealing with what medical humanities are or should be) provides several understandings of medical humanities and no broad agreement on definitions, demarcations or gains exists. Positioning subdivisions of traditions in the field, Scott-Fordsmand [9] lists three major branches referred to in the meta-literature of the field: a pedagogical/empathy-focused branch of Medical Humanities (humanities have a utilitarian, supportive function), Critical Medical Humanities (humanities have a disruptive, critical function) and Health Humanities (humanities have a function of broad inclusion within health and social care, education and research). Whilst acknowledging, in line with Scott-Fordsmand, that any boundaries made within this field are blurred, this paper focuses on the first, the pedagogical/empathy-focused branch of medical humanities, or on what Bleakley [23] describes as “…education for empathy or tolerance of the ‘other’” (p. 960). This approach was integrated into medical teaching programs in North American medical schools during the first decades of the twentieth century to counter the scientification of medicine and what had become a too narrow and reductive focus on medicine as biomedicine [9, 23, 24]. As stated by Shapiro et al. [25] “an important goal of medical humanities is to reconceptualize health care, through influencing students and practitioners to query their own attitudes and behaviors, while offering a nuanced and integrated perspective on the fundamental aspects of illness, suffering and healing” (p. 192). Using alternative teaching methods, concepts and content from the humanities to increase students’ self-reflection and their communicative abilities, the pedagogical/empathy-focused medical humanities is an interdisciplinary field concerned with understanding the human condition of health and illness in order to create sensitive health care providers, enhance empathy and to construct professional ethical values [10, 12, 26, 27]. In most countries, medical students thus experience being exposed to values, knowledge, and teaching methods during their education (e.g., through literature (or other arts subjects such as reflective writing, drama, film or music), philosophy, ethics, health psychology or person-centred approaches to communication), reflecting different epistemologies [8, 22, 28]. However, the way the epistemologies deriving from the medical humanities have been integrated into the positivist framing of the biomedical and natural sciences continues to be critiqued and debated [21, 29–31]. In the medical educations the traditional dichotomies between the humanities and natural sciences are seen reflected in discursive antithetical constructions such as the “soft” (warm-hearted) versus the “hard” (intellectually rigorous) subjects [10, 22] and the often-used dualistic sorting of medical knowledge into two main categories: objective (disease, biomedical, curing) and subjective (illness, humanities, caring) [28, 29, 32].

Referring to quantitative research findings showing that in many (but not all) medical educational contexts in the world, medical students’ self-reported empathy declines as their education progresses [33] scholars point to powerful curricula biases and other hidden influences at the level of organizational structure and culture, affecting how students prioritize the medical humanities and the amount of time invested herein [34–36]. Empirical research confirms this tendency by showing that despite adding medical humanities to the undergraduate medical curriculum, committed to teaching the value of empathic- and compassionate care, medical students experience that socialization processes within their educational environment teaches them something very different: that emotional detachment, objectivity and value neutrality are key attributes of a competent and professional physician [28, 37–40].

The above research findings point to strong influences on medical students’ identity formation and connected hereto, on how they perceive the role of the medical humanities in relation to the medical education. Understanding medical students’ perceptions of their teaching in medical humanities and how they navigate through formal and informal influences within their learning environment is important to gain insight into the depth, context and impact of the inclusion of the medical humanities and to provide contextual knowledge to the findings of some of the above-mentioned studies reporting a declining development of medical students’ empathy. The current study thus explores Danish medical students’ understanding of and reflections on how the medical humanities relate to their education, including the clinic.

## Methods

### Educational context

Medical education in Denmark is university-based, standardized to last 6 years and divided into a three-year bachelor medical education and a three-year graduate medical education. Generally, the bachelor comprises basic biomedical science courses in the pre-clinical years (1^st^ and 2^nd^ year) and clinical clerkships towards the end of the bachelor (3^rd^ year), continuing in the graduate medical education with an increase in clinical student participation -and responsibility.

### Medical humanities

The teaching content -and volume of the medical humanities vary slightly across the medical educations in Denmark. A review of the syllabi of the four Danish medical educations shows that the medical humanities are included primarily in the core bachelor curriculum as compulsory courses. Courses include health psychology, medical ethics, philosophy of science, communication and narrative medicine (at one medical school a compulsory course, at others elective). Most of these courses comprise relatively few ECTS points (European Credit Transfer System) compared to the science courses and are assessed through passing/non-passing examinations. Communication teaching, using simulation and actors to educate for sensitivity, is generally placed towards the end of the bachelor, encompassing theoretical input and practical training through simulation, continuing as pre-clinical preparation and post-clinical supervision through the graduate medical education.

### Recruitment

An announcement summarizing the study and inviting medical students to participate was posted on university web sites and posted in Facebook groups for medical students from the four medical schools. We did not set up any inclusion/exclusion criteria, e.g., demographic variables, but used a convenience sampling [41]. All students who responded to the invitation were forwarded an information letter, detailing the objectives of the study, what it entailed to participate in an interview, and information on the collection and processing of the data (complying with the General Data Protection Regulation (GDPR)). All students gave verbal and written consent and were informed that participation in the study was voluntary.

### Participants and data collection method

The medical students who participated in this study came from all four medical schools (and universities) in Denmark. Twenty-three medical students across years of curriculum and medical schools participated (see Table 1 for an overview of participants). The majority of students were from the medical schools of University of Southern Denmark (SDU) probably due to local advertisement of the research study.

**Table 1:** Overview of participants

Interviews	Number of participants	Sex		University				Year	
Students	23	Female 16	Male 7	SDU 13	AU 4	KU 5	AAU 1	1.-3 7	4.-6 16

The interviews were conducted from December 2020 to March 2021 by EAH, a senior qualitative researcher and associate professor with a background in sociology, and CVT, a junior researcher and psychologist. Because of the corona pandemic and lockdown periods, all interviews were conducted via video (Zoom) and lasted approximately 60 min. We did not find that conducting the interviews via video compromised the quality of the data, e.g., through technological challenges, lacking rapport building and/or attention to interaction dynamics [42]. Students expressed that they were used to interacting through online platforms due to online lectures and daily social interaction. We were following a semi-structured interview guide that was structured in the following overall themes: Students’ understandings of empathy, inclusion of empathy in the medical education, influences on empathy during the education, extent of empathy in the clinical context, perceived consequences of empathy. Examples of interview questions are: Could you describe how the humanities subjects are integrated into your medical curriculum? How is empathy being addressed during your education, e.g., by your peers, teachers, and clinicians? Which educational processes do you perceive to enhance and inhibit empathy and patient-centred, empathic communication in the clinic? Adjustments were made to the interview guide during the period of data collection as students’ narratives led to a further contextualization of some of the interview questions. For example, since no explicit teaching in empathy existed across medical schools, students referred to all the courses that were not “core” biomedical (narrative medicine, health psychology, communication, ethics and philosophy) and that the students thought were supposed to teach them about how to think “otherwise” about medicine, how to become patient-centred (versus illness-centred), and how to become empathic physicians. Interviews were audio-recorded and transcribed verbatim by a student assistant concurrent with the data collection.

### Analysis

A qualitative thematic analysis (TA) was performed, following the procedures described by Braun and Clarke [43, 44]. Initially, EAH and CVT listened to the audio files, and read the transcripts carefully several times, taking notes along the way. In a next step, EAH and CVT performed an open, inductive coding in NVivo software (version 12), developing codes that were grounded in the data. Codes were discussed and compared through weekly meetings. A third analyst and junior researcher, AU, with a background in nursing and health sciences, read all transcripts and was introduced to the codebook developed by EAH and CVT. Based on several discussions and readings of the existing literature, dealing with the positioning and different articulations of medical humanities, EAH and AU synthesized codes into themes and subthemes (see Table 2 for an overview of codes, subthemes and themes. The two co-authors, CMA (psychologist and researcher) and JS (general practitioner and researcher), were furthermore involved in analytic discussions along the way. The analysts were reflexive about their theoretical and epistemological positioning, discussing how these influenced the way they were analysing the data, for instance how their professional backgrounds (within sociology, psychology, nursing, general practice, and humanistic health research) might have biased them towards seeing certain patterns in the data material. That said, the analysts considered their diverse backgrounds a strength as they served as a basis for critical discussion of the status quo.

**Table 2:** Overview of themes, subthemes and codes

Themes	Subthemes	Open codes
The role of the medical humanities in the preclinical educational system	Students prioritize biomedical knowledge building The educational institution downgrades the medical humanities	Learning about empathy Not enough or good enough Narrative medicine Health psychology Communication teaching The professional versus the soft Under-prioritized by the students How empathy is addressed Empathy is a buzzword, but no real attention The gap Heavy educational pressure Result versus process Lack of time Meeting the patient as a human being Maturation process
The role of the medical humanities in the clinical educational system	The health care system undermines the medical humanities Labor division and identity formation	The clinic Lack of time Showing lack of empathy Not enough focus on empathy Structure Distance-cynicism Nurses versus doctors Out-sourcing of empathy Development in empathy Patient needs Immunization Specialties and empathy

## Results

### Theme 1: The role of the medical humanities in the preclinical educational system

What was clear from the students’ accounts, demonstrated below through empirical examples, was that the medical humanities, intended to implicitly communicate empathy as an important physician attribute and value, played a relatively minor role in the preclinical medical education, both personally in what the students themselves were motivated to learn and structurally in the way the medical humanities were delivered and prioritized.

#### Subtheme 1a) Students prioritize acquiring biomedical knowledge

Students talked about being first and foremost motivated to acquire knowledge about the healthy and the sick body and how to cure diseases, anticipating that basic natural sciences would constitute the core of the medical education: “When you study medicine, you want to learn about diseases and health. So, that is what I primarily expected when I entered the education. Not to become a psychologist or something like that”. (Student 8, 4^th^ year) The students were aware that to reach the anticipated learning goal, memorizing and processing large amounts of biomedical information was necessary: “There is, after all, a lot of knowledge that has to be crammed into our heads during these 6 years”. (Student 15, 1^st^ year).

Consequently, the courses that were not perceived to be “core” biomedical, e.g., communication courses, were not dedicated sufficient time and energy:

I think, after having attended the courses [communication courses] that it’s okay. But I just think that it came at a time when you feel that there are so many other things. And then you are more inclined to emphasize the professional [biomedical] part. So, I’d rather concentrate on that, instead of all the "soft" and not so easily definable subjects. Because you kind of get the thought: How relevant is this actually to my education? (Student 5, 5^th^ year)

I mean, when we are under pressure with anatomy or biochemistry, then this is where you choose to have your focus. (Student 9, 4^th^ year)

Facing a pressure to learn and master biomedical science knowledge ahead of frequent examinations, the students became conditioned to rationalize strategically: “I mean, right now we have anatomy, and there I must quickly cut away, so what is important and what’s not in relation to the exam? And then I must learn what is important, otherwise I won’t get a good grade.” (Student 17, 1^st^ year).

The students’ learning activities were thus in large part driven by attaining sufficiently high-performance goals to persevere, prioritizing outcome over process. As a consequence, the medical humanities courses, entailing whole other ways of learning, one of open curiosity and reflection, could be experienced as disturbing elements: “During these courses you kind of think: argh, is it necessary for me to spend time on this? I'd rather study for my exams.” (Student 21, 6^th^ year).

One student, talking about the students’ tendency to neglect health psychology, voiced regret that extrinsic motivators were controlling the behavior of himself and his peers since, as he acknowledged, the subject was “actually super important”:

We have this course called health psychology, and it’s a course that is being neglected. It’s a course that is not so hard to understand and I think – because the other subjects are so heavy and hard - the subjects that are perceived as easy and soft just become something that just needs to be passed. So, you don’t show up at class very often, and you take everything a little lightly. That’s kind of the general attitude or approach to it...but I do hear a lot of people, including myself, saying that health psychology is actually a super important subject, but you just cannot bother using time on it, if you are only required to pass the exam. (Student 10, 5^th^ year)

As seen in the above excerpts, health psychology, described as “easy”, “soft”, “something that just needs to be passed”, but also “important”, is perceived to compress an already crowded curriculum, wherefore it is being attributed a minor role by the medical students, and seen as peripheral to the core learning objectives of the bachelor medical education.

#### Subtheme 1b) The educational institution downgrades the medical humanities

When asking students to reflect on the role attributed by the medical institution to the medical humanities, they described what they perceived to be a small institutional focus, despite recent inclusion of medical humanities courses in the curriculum: “Well, narrative medicine has been implemented, but it’s not my impression that they [the medical humanities], are being prioritized in the medical education.” (Student 15, 1^st^ year) In relation to teaching empathy in specific, and the curricula role of empathy-enhancing teaching, a student explained: “I have not heard the word empathy mentioned many times, I think. I have never really had the impression that there has been so much focus on empathy in our teaching.” (Student 4, 5^th^ year) Yet another student commented on how empathy figured as a” buzzword” in the educational environment, i.e., a word which students were socially and academically conditioned to mention without putting real value or intrinsic motivation into it:

It's kind of like: “oh yes, of course we have to be empathic”, like a buzzword you learn. And we’re not getting tested in it... and there is not much talk about it. And the reason for this is that we focus primarily on the enormous amount of biomedical knowledge we need to gain and the focus on being as accurate and correct as possible all the time with our medical terminology. (Student 20, 6^th^ year)

The impression that the medical humanities were only attributed a minor role in the medical curriculum was further strengthened by the structural conditions under which they were delivered: either lacking assessment or assessed on a pass or fail scale, comprising few ECTS and positioned between high-ECTS biomedical courses. e.g., anatomy and physiology. Although students recognized that assessing empathy and other “soft” interpersonal skills could be challenging, the students detailed the unintended consequences of making low-stake assessments and low-ECTS courses, namely minimal time -and effort investment. As two students explained in relation to a narrative medicine course:

Because it was a relatively small course of 2 ECTS points or something like that. So, you only read the short stories to be able to follow the analysis during class, but you don’t spend 10 hours analyzing them at home. We spend our time on the professional [biomedical] part. (Student 9, 4^th^ year)

We are not being examined in these subjects. Which is why people choose not to spend time on it. Because it’s not what is important to our result. (Student 17, 1^st^ year)

The above excerpts point to a direct relation between the institutional valuation of the humanistic knowledge-building (formal and informal), curricula structure and delivery, and the students’ own motivation and effort invested in these subjects.

### Theme 2: The role of the medical humanities in the clinical educational system

#### Subtheme 2a) The health care system undermines the medical humanities

Several students, while talking about the health care system as their future workplace, and their personal experiences from their clinical training, referred to a health care system characterized by structures and values that were challenging the provision of person-centred, empathic care. As perceived by the students, a predominant atmosphere of time pressure and other stressors, resulting from a high patient volume, staff shortages and documentation requirements influenced the way clinicians managed patient encounters. Students oftentimes observed physicians behaving rushed and showing lack of empathy: “The doctor, he was just busy running through routine procedures. He was being nice, surely—but he didn’t articulate, at any point in the conversation, that it was evident that she [the patient], was on the verge of crying.” (Student 7, 3^rd^ year).

Another student summarized her experiences from the clinic thus:

When I have been taken by the hand by someone in the health care system, I experience that they attach great importance to the medical work itself. "Find a diagnosis" or "find the cure", "find the right treatment". There is very little time to get a sense of the patients. You don’t pay too much attention to whether a patient is crying, twisting his hand, or somehow expressing that they’re under pressure. Or happy. Or angry. And I regard it as an extremely dangerous trend. That you downplay the human being, the patients you meet, and then put them in mechanical professional boxes, and then you do not necessarily catch all the soft stuff, compassion - the things that are said between the lines. (Student 1, 5^th^ year)

The above excerpt indicates that a rushed and time-pressured environment counteracts with the empathic and person-centred care that the medical humanities are committed to teach. Recognizing how organizational structures forced an unemphatic behavior upon health professionals, transforming at the same time their beliefs about how to be a physician, was a source of frustration and conflict as explained by a student in the following excerpt:

And it’s often a matter of time pressure or staff shortages or... I’m really sad and frustrated that this part gets to dictate how much time you actually end up having for the things that you consider important in a medical interview. (Student 18, 6^th^ year)

Reflections about conflicting values and messages as well as evasion of responsibilities on a macro level in respect to creating favorable structures for humane care in hospital settings were made by one student:

It’s easy for an administrative management to say: “Remember empathy!” if there’s not time for any of that in the entire health care system. Well, you know...it’s just so easy administratively to relinquish responsibility and say: “Remember – the patient first!”, where I think: "well, then you have to change the structures of the system!" You can’t hold the individual doctor responsible. And I've seen a lot of junior doctors who are very nice and really want the best for their patients, but the framework simply does not allow it. (Student 5, 5^th^ year)

As we shall see further unfolded in the next section, students came to realize through their observations and participations in the clinic that in fact empathic care is not supposed to be part of their professional areas of expertise, since empathy was oftentimes seen as something that was being outsourced to the nurses.

#### Subtheme 2b) Work task distribution and identity formation

During clinical clerkships, a pivotal time in which students begin their transition from students to physicians, students experienced missing alignment between the human values inherent in the medical humanities, such as empathy and compassion, and those that dominated the clinic. Furthermore, students gradually came to understand that these values were in fact distributed within the medical team such that by and large the physicians were paid to accomplish one set of tasks and the nurses another. A recurring observation made by the students during clinical clerkships was that “nurses were better than physicians at showing empathy towards patients” because they were paid to spend more time with patients, getting to know them as “persons” in contexts. In the following excerpt a student describes his observations of how work tasks are distributed between physicians and nurses:

The empathy work, all the caring, it’s primarily the nurses’ job. The nurses know the patients and have daily conversations with them and watch them develop as persons. I know for a fact that many doctors, upon delivering important and serious messages to the patients, sit with them for a while, and the nurse takes part in this, and the patients are allowed to ask all the questions they want and to process the information a little. And when this is over, the doctor leaves the room. And then the nurse stays to take over... And again, it’s all about time: the nurse has been given this job and the time for it. So, in this way, we outsource empathy. (Student 10, 5^th^ year)

Apart from learning how empathy was being “outsourced” to nurses, implying that the medical humanities’ ideal of “empathic and holistic care” was largely held in one group of professionals, students also learned how empathy was largely held in one domain of their future medical practice: the diagnostic processes where they had been taught how to use their senses, relating sensitively and empathically to patients, to complete their distributed work task effectively:

And this is also one of the things we learn – like making sure that you effectively listen to the patient and discover what concerns they have and what they fear. And then quickly get it summed up so that you can get on to the actual medical examination and then just summarize: “This is what we know – and this is the plan”. And then you can do it really effectively. (Student 20, 6^th^ year)

Experiencing these work task distributions, students gradually came to redefine their perception of which values were tied to their future profession and which functions were “part of the job”:

I had thought that the job as a physician included more care. So quite specifically, a lot of times you go see a patient, and if there is something else, besides the one problem you need to focus on, you ring the bell and get the nurse to come in and take over. And this is not necessarily a bad thing, but I would have thought it great if you had time to talk with the patients. But again, that’s not our job so... (Student 10, 5^th^ year)

Refuting this compartmentalization, a student commented: “In fact, there is no reason why one of them should show more empathy than the other one. Because both see sick people.” (Student 7, 3^rd^ year) However, pressured to meet performance indicators, documentation, time management and efficiency the physician’s incentives to practice empathic care was perceived as minimal, shaping students’ behavior and identity:

We can’t write in a medical journal that we’ve been so-and-so empathic, that we’ve listened so-and-so much or talked so-and-so much about their concerns... because suddenly that’s not what’s important anymore, if you know what I mean? Instead, it’s important to write that you have done a specific thing and that you have tested for this and that so that we cover our own ass. And somehow, you suddenly care more about those numbers and demands instead of prioritizing being emotionally present and showing empathy towards the patient. (Student 11, 5^th^ year).

The student in the above excerpt describes the process of increasingly internalizing values, norms and imperatives of the clinic into one’s professional identity to fit system rationales, and how the lacking empathy incentives of medical practice, e.g., journal writing, might make a physician choose to conduct him/herself strategically instead of empathically.

## Discussion

This qualitative study provides knowledge about Danish medical students’ reflections on and experiences with the inclusion of the medical humanities into their medical education, including how they perceive their role and relevance in the pedagogical environment in the preclinical years and in clinical rotations. Overall, what Bleakley and Marschall have named “weak inclusion” [45], referring to an inclusion of the medical humanities in medical curricula that in fact leads to the very divide that the medical humanities seek to bridge, can arguably be said to apply to the present Danish medical educational context. Lacking institutional conditions and structures to promote a “strong” inclusion of the medical humanities, our findings show that students internalize epistemic dichotomous views of the divide between the “hard” and “soft” teaching subjects as part of a professional identity formation.

By the same token, showing how students have a preconceived understanding of what skills and values the physician role encompasses, our findings suggest that a one-sided biomedical identity-formation starts even before entering the university. Students conceive of the medical curriculum as consisting first and foremost of biomedical science knowledge, an image that is further strengthened upon entering the undergraduate, medical education, where the medical humanities are diminished as surplus to the core study.

Upon entering the clinical curriculum the values that the medical humanities are committed to cultivate in future physicians are in large part being undermined by an antithetical culture of performance, productivity and -efficiency pressures. As part of this tendency, time management and work task distribution routines in the clinic exist, leading to a controlled and disciplined care environment that by and large deprive medical students and physicians of providing empathic care. Our findings thus point to a fundamental contradiction in medical education: that students, through the introduction of the medical humanities components in the preclinical years are encouraged to become empathic, compassionate and person-centred physicians but that these values are overshadowed by values such as emotional insulation and objectivity. These findings confirm arguments within existing research [39, 46], namely, that communication of values on a subtle and hidden level influence students’ valuation of humanistic approaches to medicine, giving rise to personal value conflicts and dilemmas in students who find it difficult to navigate in an educational terrain representing the formal and the informal (hidden) curriculum.

All the above emphasizes the extent to which a piecemeal or partial introduction of medical humanities, that focuses more on content (syllabus) than on process (the making of identity) is never going to have much effect [47]. Extending the body of literature that already exists on medical humanities in the medical education the above findings suggest that the Danish medical curricula need to be reconceptualized (both in the preclinical medical school pedagogical environment and in the clinical years) such that the medical humanities are incorporated into the core curriculum through biomedical science and clinical lenses, focusing on how the humanities have a fundamental role to play in medicine. Elaborating on this reconceptualization, Scott-Fordsmand [9] argues for establishing a bidirectionality of the medical humanities such that medicine does not just passively receive knowledge from the humanities (movement from humanities to medicine) but actively contributes with insights into a humanities context (movement from medicine to humanities), hereby emphasizing the double epistemic nature of medicine. For this to happen, as argued by Bleakley [23, 47], the medical humanities must be planned as a pedagogical challenge in which the contextual factors behind anatomy and biomedical sciences are taught by science teachers and anatomists through aesthetics, ethics and politics and given as high a profile as instrumental values. Furthermore, clinicians as educators should communicate to students that the medical humanities can be used as resources in clinical work, focusing upon the appreciation of sensibility, tolerance of ambiguity and epistemic uncertainty in medicine [47].

The study findings thus stress, in line with what has been argued elsewhere [48], that exercising empathy in health care depends not only on the individual (student or health professional) but also on the medical school and clinical pedagogical environment in which the individual is embedded. The educational system, including the health care systems, have a huge role to play in facilitating or impeding individual efforts or value-based behavior wherefore the locus of attention should be directed, not only towards the individual student, but to the learning conditions established in the educational environments that promote or hinder the creation of caring health care systems.

This is also the key message conveyed by The Danish Council on Ethics that has recently published its annual report and issued a statement about “Care in the health care system”. What both documents wish to address is the conflicting relationship between the need for human care, including empathy, and the pursuit of greater economic efficiency of today’s health care system [49]. Furthermore, it is emphasized that care should never just be seen as the responsibility of the individual but institutionally ensured to facilitate the formation and preservation of morally justified caring actions. As emphasized in reports following scandals and high-profile cases in the UK, the pressure to meet efficiency and operational targets not only endangers positive outcomes of the patients but also the psychological well-being of health care professionals [50]. Thus, the weak inclusion of the medical humanities in the medical educational system, that this study has sadly identified, poses a major problem for future clinical care and health professionals’ well-being. To enhance a strong inclusion of the medical humanities, and on the basis of our analysis, we suggest continuing working on:

-reconstructing the “image” and identity of the medical education to also encompass holistic and humanitarian formation- and care. In connection herewith, the educational system would need to reassess its priorities and structures to eliminate dehumanizing tendencies.

-establishing a “positive hidden curriculum” [51], meaning that medical humanities are situated as an integrated part of science courses and clinical training. As integrated medical humanities are given more weight (increase the number of ECTS) and are included in “high-stake” assessments equal to those of the biomedical courses.

-establishing continuity and value coherence between the pre-clinical and clinical years. Clinicians, acting as role models, need to know about medical humanities syllabi and through their actions reinforce the messages that this teaching is intended to convey, e.g., the benefits of empathic care in regard to diagnostic processes, adherence to treatment, satisfaction, physician well-being, etc.

-creating structures and conditions on a macro- and meso-level that incentivize empathic behavior- and practices, facilitating caring institutions that, ultimately, as research shows, creates better health care, better health among patients and professionals, health economical gains, a decrease in patient complaints, etc.

### Strengths and limitations

Providing insight into how the medical humanities serve a subordinate role in the Danish medical educational system, the results of this multi-institutional study provide contextual knowledge and depth to the findings on the longitudinal development of medical students’ empathy. For despite being limited to a Danish medical educational context, the findings can be transferred to other similar educational settings with similar educational set-ups. In terms of analytical validity, analytic collaborations between researchers with different professional backgrounds, increased the trustworthiness of the findings.

However, our study has some relevant limitations. First, since we were using a convenience sampling, the number and the sex of the participating medical students were not evenly distributed across the four medical schools which means that the findings primarily capture learning experiences from students attending three out of the four included medical schools. However, we assess that a multi-institutional study like ours leads to higher transferability to similar educational contexts than single institution studies. Second, we captured students’ learning experiences retrospectively which might have created some retrospective recall distortion.

## Conclusion

The results of this study have important implications for ongoing efforts to position and valorize the medical humanities in the field of medical education. Moving beyond a focus on students’ empathy levels (whether it declines or not), this paper proposes to focus on the values and norms of medical educational systems that are being internalized in future physicians’ identity formation and that undermine a strong and balanced inclusion of the medical humanities into medical education. To achieve a strong inclusion, the medical humanities should be incorporated into the curriculum through biomedical science and clinical lenses, focusing on how the medical humanities have a fundamental role to play in medicine.

## Data Availability

There is no public availability to the interview transcripts outside of the research team due to reasons of confidentiality. Data are however available from the corresponding author on reasonable request.
